# The recently identified modifier of murine metastable epialleles, Rearranged L-Myc Fusion, is involved in maintaining epigenetic marks at CpG island shores and enhancers

**DOI:** 10.1186/s12915-015-0128-2

**Published:** 2015-03-26

**Authors:** Sarah K Harten, Harald Oey, Lauren M Bourke, Vandhana Bharti, Luke Isbel, Lucia Daxinger, Pierre Faou, Neil Robertson, Jacqueline M Matthews, Emma Whitelaw

**Affiliations:** Epigenetics Laboratory, QIMR Berghofer Medical Research Institute, Herston, Brisbane, QLD 4006 Australia; Department of Genetics, La Trobe Institute for Molecular Science, La Trobe University, Bundoora, Melbourne, VIC 3086 Australia; School of Biomedical Sciences, Faculty of Health, Queensland University of Technology, Brisbane, Australia; Department of Biochemistry, La Trobe University, Melbourne, VIC 3086 Australia; School of Molecular Bioscience, University of Sydney, Sydney, NSW 2006 Australia; Present Address: Center for Human and Clinical Genetics, Leiden University Medical Center, Leiden, The Netherlands

**Keywords:** Rearranged L-Myc Fusion, Rlf, DNA methylation, Enhancers, Bisulphite

## Abstract

**Background:**

We recently identified a novel protein, Rearranged L-myc fusion (Rlf), that is required for DNA hypomethylation and transcriptional activity at two specific regions of the genome known to be sensitive to epigenetic gene silencing. To identify other loci affected by the absence of Rlf, we have now analysed 12 whole genome bisulphite sequencing datasets across three different embryonic tissues/stages from mice wild-type or null for *Rlf*.

**Results:**

Here we show that the absence of Rlf results in an increase in DNA methylation at thousands of elements involved in transcriptional regulation and many of the changes occur at enhancers and CpG island shores. ChIP-seq for H3K4me1, a mark generally found at regulatory elements, revealed associated changes at many of the regions that are differentially methylated in the *Rlf* mutants. RNA-seq showed that the numerous effects of the absence of Rlf on the epigenome are associated with relatively subtle effects on the mRNA population. *In vitro* studies suggest that Rlf’s zinc fingers have the capacity to bind DNA and that the protein interacts with other known epigenetic modifiers.

**Conclusion:**

This study provides the first evidence that the epigenetic modifier Rlf is involved in the maintenance of DNA methylation at enhancers and CGI shores across the genome.

**Electronic supplementary material:**

The online version of this article (doi:10.1186/s12915-015-0128-2) contains supplementary material, which is available to authorized users.

## Background

We recently reported that *Rearranged L-myc fusion* (*Rlf*) acts as an epigenetic modifier [1]. The gene emerged from a sensitized N-ethyl-N-nitrosourea (ENU) mutagenesis screen carried out to identify factors involved in epigenetic regulation of transcription. The screen was based on the identification of mice with altered expression of a multi-copy GFP transgene that is susceptible to epigenetic gene silencing [2]. This is a dominant screen and we call the mutant lines *Modifiers of murine metastable epiallele Dominant (MommeD)* [2]*.* We found that three of the lines, *MommeD8*, *MommeD28* and *MommeD34*, carry mutations in *Rlf* and have designated the alleles *Rlf*^*MommeD8*^, *Rlf*^*MommeD28*^ and *Rlf*^*MommeD34*^. Of these, *Rlf*^*MommeD28*^ and *Rlf*^*MommeD34*^ are null alleles and *Rlf*^*MommeD8*^ is hypomorphic [1]. Mice heterozygous for the mutant alleles displayed increased silencing of both the reporter transgene and another epigenetically sensitive allele, *agouti viable yellow* [1]. Mice heterozygous for *Rlf* mutations are viable, with no overt abnormalities. Mice homozygous for the null alleles die around birth. Little is known about the function of Rlf, although the predicted presence of 16 widely-spaced zinc fingers suggests a role in transcription [3]. Bisulphite sequencing at the reporter transgene revealed increased DNA methylation in late gestation *Rlf*^*MommeD28/MommeD28*^ embryos, consistent with its reduced expression [1].

To discover whether or not Rlf has functions at other loci, we have carried out whole genome bisulphite sequencing at three different stages of development and provide evidence that Rlf has a role in the maintenance of DNA hypomethylation at thousands of elements across the genome. These regions overlap with those previously found to be differentially methylated across different tissue types, called tissue-specific DMRs (tsDMRs) [4-8]. Tissue-specific DMRs overlap with elements involved in transcriptional regulation, in particular enhancers.

Here we show that Rlf has a role in maintaining DNA hypomethylation at enhancers across the genome. Increases in DNA methylation that occur in the absence of Rlf, are accompanied by reductions in H3K4me1 occupancy.

## Results

### Loss of Rlf results in an increase in DNA methylation at short lowly methylated regions across the genome

To understand how Rlf affects DNA methylation at a genome-wide level, we carried out genome-wide bisulphite sequencing on the livers of E14.5 embryos (*Rlf*^*+/+*^ and *Rlf*^*MommeD28/MommeD28*^). We chose E14.5 liver because the original screen was carried out using erythroid cells and the liver is the major erythropoietic tissue at this stage of development. Also, since E14.5 embryonic liver is one of ENCODE’s chosen tissues, associations can be made between sites of differential DNA methylation and histone marks. Mice homozygous for the null allele appear grossly phenotypically normal at this stage, reducing the risk of possible changes to DNA methylation that are the consequence of their demise at later developmental stages. Bisulphite sequencing was carried out on E14.5 livers from two wild-type (*Rlf*^*+/+*^) and two homozygous (*Rlf*^*MommeD28/MommeD28*^) littermates to an average depth of 30-fold per sample. Approximately 85% of the CpGs in the mouse genome were covered by >5 reads and approximately 75% were covered by >20 reads (Additional file 1: Figure S1). For each CpG, the percentage of converted and unconverted Cs across all reads was calculated (%mCG). Sequencing of unmethylated lambda DNA, spiked into each sample, confirmed complete conversion (>99%). Regions differentially methylated between the wild-type and homozygous samples were identified using the following parameters: the region must contain >10 CpGs and each CpG must be covered by >5 reads. A call was made if the difference between the %mCG of the wild-type (average of two) and homozygous (average of two) samples was greater than 15%. Use of these parameters might bias the analysis to CG rich regions.

**Figure 1:**
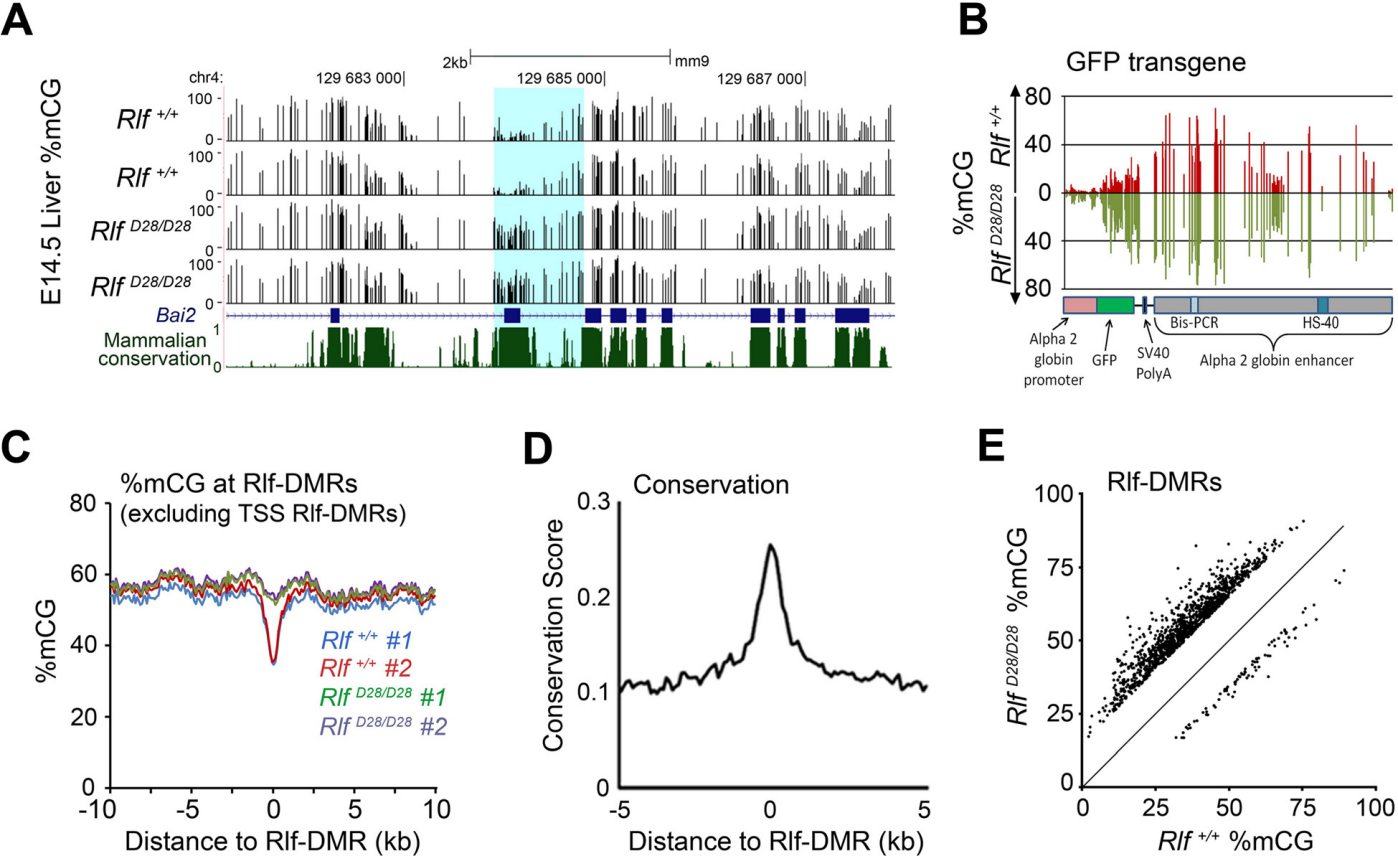
**DNA methylation is increased at ~ one thousand loci in the genomes of** ***Rlf*** ^***MommeD28/MommeD28***^ **mice. (A)** A UCSC Genome Browser snapshot showing a representative Rlf-DMR, within the gene *Bai2,* with increased methylation in *Rlf* ^*MommeD28/MommeD28*^ compared to *Rlf* ^*+/+*^ mice. The gene’s exons are indicated by blue boxes, introns by connecting lines and direction of transcription by the intronic arrows. **(B)** The mice carry a GFP transgene used in the ENU mutagenesis screen that identified Rlf as an epigenetic modifier [1]. Bisulphite sequencing reads covering the transgene show hypermethylation of DNA from *Rlf* ^*MommeD28/MommeD28*^ mice throughout its length. Transgene sequence features, and a region previously targeted by bisulphite PCR [1], are indicated. **(C)** The average methylation of regions differentially methylated in *Rlf* ^*MommeD28/MommeD28*^ mice, compared to wild-type, are plotted. Only Rlf-DMRs >2.5 kb from TSS were included (n = 1,074). **(D)** Average PhastCons scores for placental mammals at Rlf-DMRs. **(E)** Scatterplot of average CpG methylation observed in *Rlf* ^*MommeD28/MommeD28*^ mutants compared to wild-types for 1,329 Rlf-DMRs identified in E14.5 liver.

We found 1,329 differentially methylated regions across the genome and call these Rlf-DMRs (Additional file 2: Table S1). Hierarchical clustering of the Rlf-DMRs showed that the two biological replicates of each genotype clustered together, attesting to the reproducibility of the technology (Additional file 3: Figure S2). A representative screen shot is shown (Figure 1A). One of the Rlf-DMRs was the multicopy reporter transgene, the expression of which had been used to identify the *MommeD* mutants in the original mutagenesis screen. This is consistent with our previous findings following bisulphite PCR of a small segment of this element [1]. The Rlf-DMR extended across the 5 kb transgene, including the HS-40 enhancer region (Figure 1B).

In general, the Rlf-DMRs were short (~1 kb), occurred at regions of the genome that are less methylated than the surrounding DNA (Figure 1C) and overlapped with regions that are conserved in placental mammals (Figure 1D). These characteristics are consistent with those reported for tissue-specific differentially methylated regions (tsDMRs) [5]. Comparison of the two datasets showed that 59% of the E14.5 liver Rlf-DMRs overlapped with a tsDMR (data not shown). The failure of some to overlap is likely to be a reflection of lower coverage in the data used to identify tsDMRs [5]. Most of the Rlf-DMRs, (n = 1,246; 94%) were more methylated in the mutants, consistent with our previous findings at the transgene locus (Figure 1E) [1].

### Rlf–DMRs overlap with elements involved in transcriptional regulation, including those at exons

**Figure 2:**
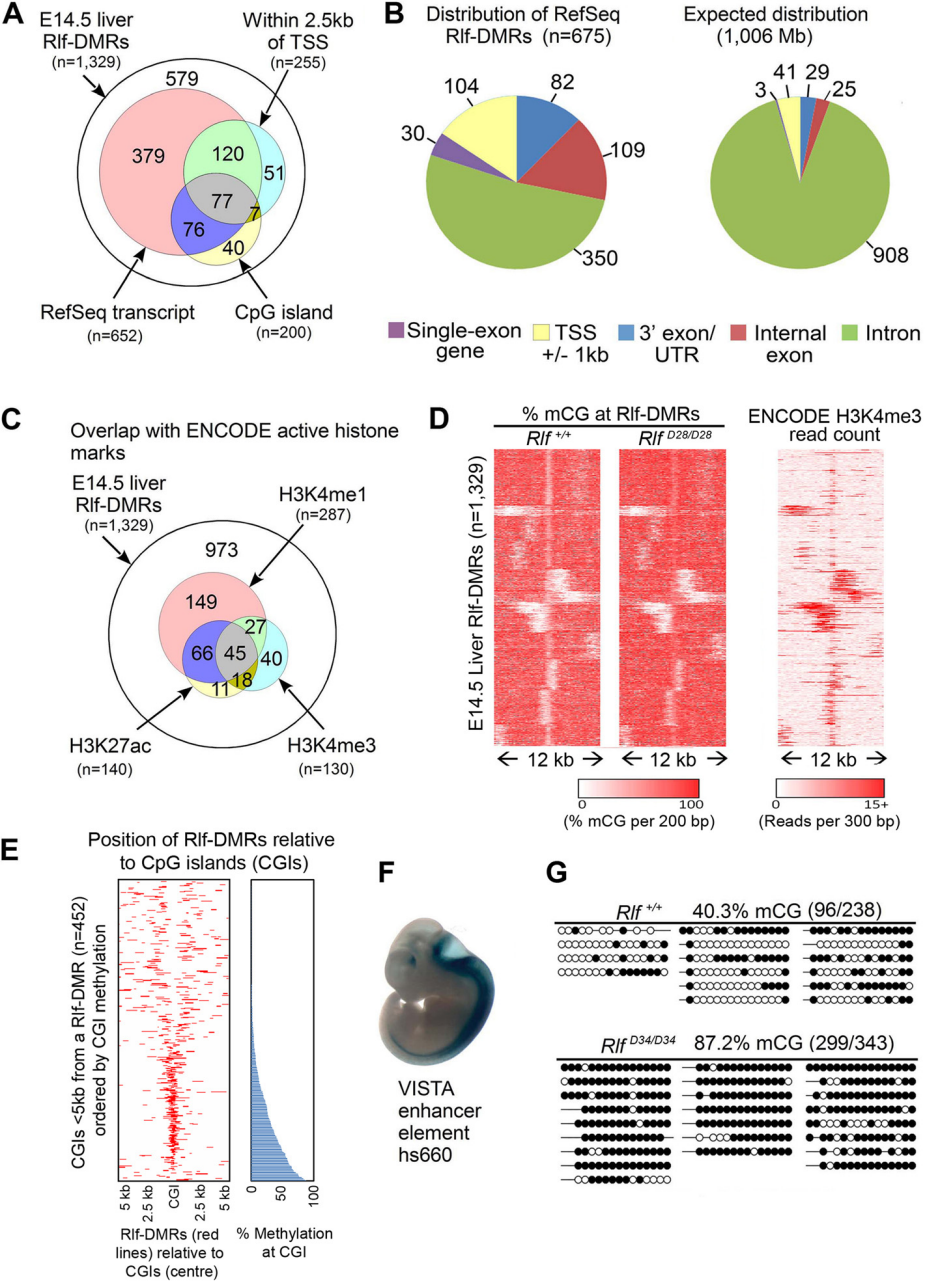
**Rlf-DMRs overlap with regulatory regions. (A)** E14.5 liver Rlf-DMRs were investigated for overlap with RefSeq genes, proximity to TSS and CpG islands. **(B)** E14.5 liver Rlf-DMRs overlapping RefSeq transcripts, were classified according to overlap with transcript features. The central CpG dinucleotide of each Rlf-DMR was used to define the overlap. Rlf-DMRs that overlapped multiple features were assigned to a single feature according to following ranking: TSS > single exon transcripts > 3′ exon/untranslated region (UTR) > internal exon > intron. The expected distribution was defined as the union of each feature category genome-wide, subtracting overlapping features of higher rank (1,006 Mb). **(C)** Counts of E14.5 liver Rlf-DMRs enriched for the histone marks H3K4me1, H3K4me3 and H3K27ac in E14.5 liver (obtained from ENCODE) **(D)** Heat plots showing DNA methylation and H3K4me3 levels surrounding E14.5 liver Rlf-DMRs. **(E)** Plot showing position of E14.5 liver Rlf-DMRs relative to CpG Islands for Rlf-DMRs located within 5 kb of a CpG island. The data has been plotted around the centre of the CpG island and sorted according to the methylation level of the CpG island. **(F)** An example of region of DNA at a Rlf-DMR that has been shown to have enhancer properties in neural tube and forebrain of E11.5 embryos [9]. **(G)** Bisulphite PCR validation of the E14.5 liver Rlf-DMR located between *Smad3* and *Smad6*.

Of the 1,329 Rlf-DMRs identified in E14.5 liver, approximately half overlapped with RefSeq transcripts (n = 652) and half were intergenic (n = 677) (Figure 2A). A relatively small proportion of the Rlf-DMRs, 255 of the 1,329, lay within 2.5 kb of a transcriptional start site (TSS) and we found little overlap, 200 of the 1,329, with the 16,000 CpG islands annotated in the mouse genome in the UCSC Genome Browser. Together these findings suggest that a minority of Rlf-DMRs overlap with promoters, consistent with findings for tsDMRs [5] and T-DMRs [4].

Of those Rlf-DMRs that overlapped with RefSeq transcripts, a large proportion, ~50%, were at exons (including the 3’UTR) (Figure 2B). This is significantly more than would be expected based on the proportion of genic sequence that is exonic (~10% of RefSeq transcripts). Recent analysis of enhancer-specific ChIP-seq data has revealed that many exons act as enhancers, affecting transcription of either the gene in which they reside or a neighbouring gene [10]. Our results are consistent with this finding.

Using the ENCODE E14.5 liver dataset, we found that 287 of the 1,329 E14.5 liver Rlf-DMRs were enriched for H3K4me1 ChIP-seq reads (Figure 2C), confirming overlap with enhancers. Only 130 of the 1,329 Rlf-DMRs were enriched for H3K4me3, a modification more commonly associated with promoters. Many of the Rlf-DMRs with H3K4me1 peaks also had H3K27ac peaks, consistent with active enhancers (Figure 2C). Again, this is reminiscent of what has been reported for tsDMRs [5]. When a Rlf-DMR was associated with an unmethylated CpG island, the methylation change tends to occur at the boundaries, the CpG island shores (Figure 2D and E). It is important to emphasise that most of the E14.5 liver Rlf-DMRs, 973 of 1,329, were not enriched for any of these histone marks and might represent “vestigial” enhancers (see discussion below).

Some E14.5 Rlf-DMRs were found to overlap with DNA elements that have been tested for enhancer activity *in vivo* (VISTA database, [9]). In total 18 overlapped, of which 11 had been found to be positive for enhancer function. For example, a VISTA-validated intergenic Rlf-DMR that lies between *Smad3* and *Smad6* was shown to drive expression in neural tube at E11.5 (Figure 2F). The differential methylation of this Rlf-DMR was validated in E14.5 liver tissue from *Rlf*^*+/+*^ and *Rlf*^*MommeD34/MommeD34*^, using bisulphite PCR sequencing (Figure 2G).

### Loss of Rlf results in a decrease in H3K4me1 at discrete regions across the genome

**Figure 3:**
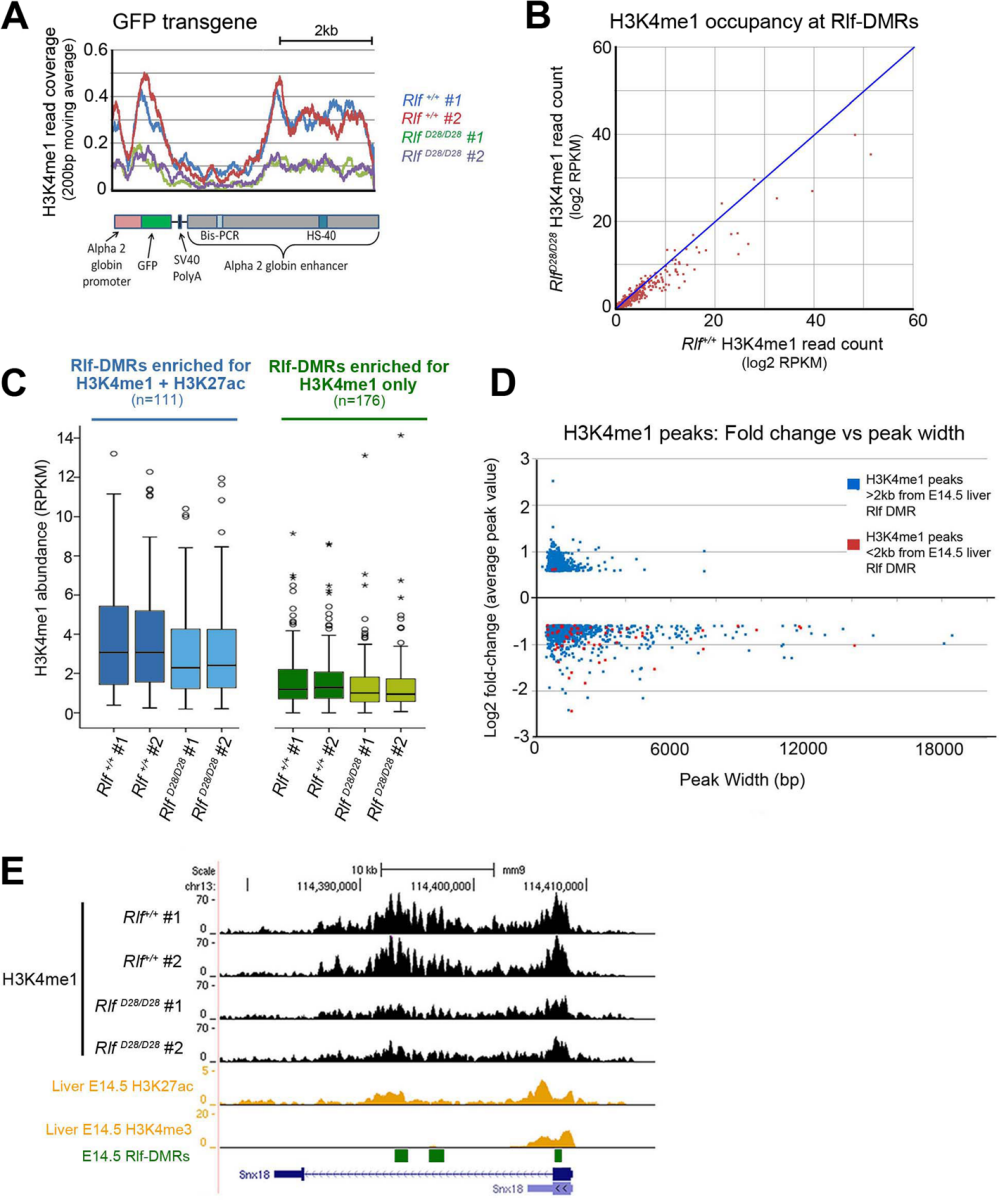
***Rlf*** **homozygous mutants show altered H3K4me1 occupancy across the genome. (A)** H3K4me1 occupancy at the GFP transgene in chromatin extracted E14.5 fetal livers of two *Rlf* ^*+/+*^ and two *Rlf* ^*MommeD28/MommeD28*^ mice. **(B)** Scatter plot showing H3K4me1 occupancy at E14.5 liver Rlf-DMRs **(C)** A box-whisker plot showing H3K4me1 abundance in *Rlf* ^*+/+*^ and *Rlf* ^*MommeD28/MommeD28*^ mice at loci overlapping either putative active or poised regulatory regions in the E14.5 liver (marked by H3K4me1 and H3K27ac or H3K4me1 only, respectively). In both cases RPKM values for the homozygous replicates are significantly different from the wild-type replicates (Mann–Whitney U test: *p* < 1×10^−8^). **(D)** Scatter plot showing fold change of peaks with >50% alteration in H3K4me1 occupancy. Red and blue dots represent peaks either within 2 kb of a Rlf-DMR or further from a Rlf-DMR, respectively. **(E)** Screen shot showing a representative region with reduced H3K4me1 occupancy in *Rlf* ^*MommeD28/MommeD28*^ mutants samples compared to *Rlf* ^*+/+*^ mice. The position of three E14.5 liver Rlf-DMRs are represented by green boxes.

To ask whether loss of Rlf also affects chromatin marks at Rlf-DMRs, we performed ChIP-seq for H3K4me1. Chromatin was extracted from two wild-type and two *Rlf*^*MommeD28/MommeD28*^ E14.5 fetal livers. For each sample, 28–40 million reads were sequenced. We saw a good correlation between our wild-type datasets and those in ENCODE (data not shown). To enable characterisation of H3K4me1 occupancy at the GFP transgene, a subset of 1x10^7^ reads were selected from each dataset and mapped to a mouse genome containing the sequence of the GFP transgene as a separate chromosome. The GFP transgene showed reduced H3K4me1 read coverage in homozygous mutants, consistent with the observed increase in DNA methylation and decrease in expression (Figure 3A). Numerous changes in H3K4me1 occupancy at Rlf-DMRs were observed (Figure 3B). Of those Rlf-DMRs with H3K4me1 enrichment, the majority showed a decrease in H3K4me1 in the mutants, consistent with that expected from the DNA methylation change (Figure 3B).

Whilst H3K4me1 marks active and poised enhancers, H3K27ac marks active enhancers and promoters [11]. To assess whether loss of Rlf specifically affects active enhancers, we divided Rlf-DMRs into two groups; those with greater than two-fold enrichment of H3K4me1 and H3K27ac and those with only the former (Figure 3C). The box–whisker plots show that in both groups H3K4me1 occupancy was significantly reduced in homozygous mutants compared to wild-types (Figure 3C). This finding suggests that at Rlf-DMRs, loss of Rlf affected H3K4me1 occupancy at both active and poised enhancers.

We were keen to see if H3K4me1 occupancy was affected at other sites in the genome. H3K4me1 peaks were identified and after combining peaks from each of the four datasets, a total of 38,689 were obtained (Additional file 4: Table S2). Analyses of H3K4me1 occupancy at these peaks revealed numerous changes in Rlf mutants compared to wild-types. Using a fold-change cut-off of 50%, 686 sites were found with increased occupancy and 856 with reduced occupancy. The majority of altered sites showed modest fold changes. Only a small subset of sites that showed H3K4me1 changes had a Rlf-DMR within 2 kb (Figure 3D; blue vs red dots). The majority of the latter were decreased, as expected. This means that many of the sites with decreased H3K4me1 occupancy did not have a Rlf-DMR within 2 kb. The width of peaks with altered H3K4me1 were generally larger than the width of Rlf-DMRs, consistent with data from others on the width of H3K4me1 peaks at and around enhancers (Figure 1C & Figure 3D) [5]. Figure 3E shows a representative screen shot of one of the regions in which H3K4me1 occupancy was found to be reduced in *Rlf* homozygous mutants.

Our results suggest that in at least some settings loss of Rlf was sufficient to induce a histone change in the absence of a DNA methylation change.

### Rlf-DMRs exist across a range of tissues and developmental stages

**Figure 4:**
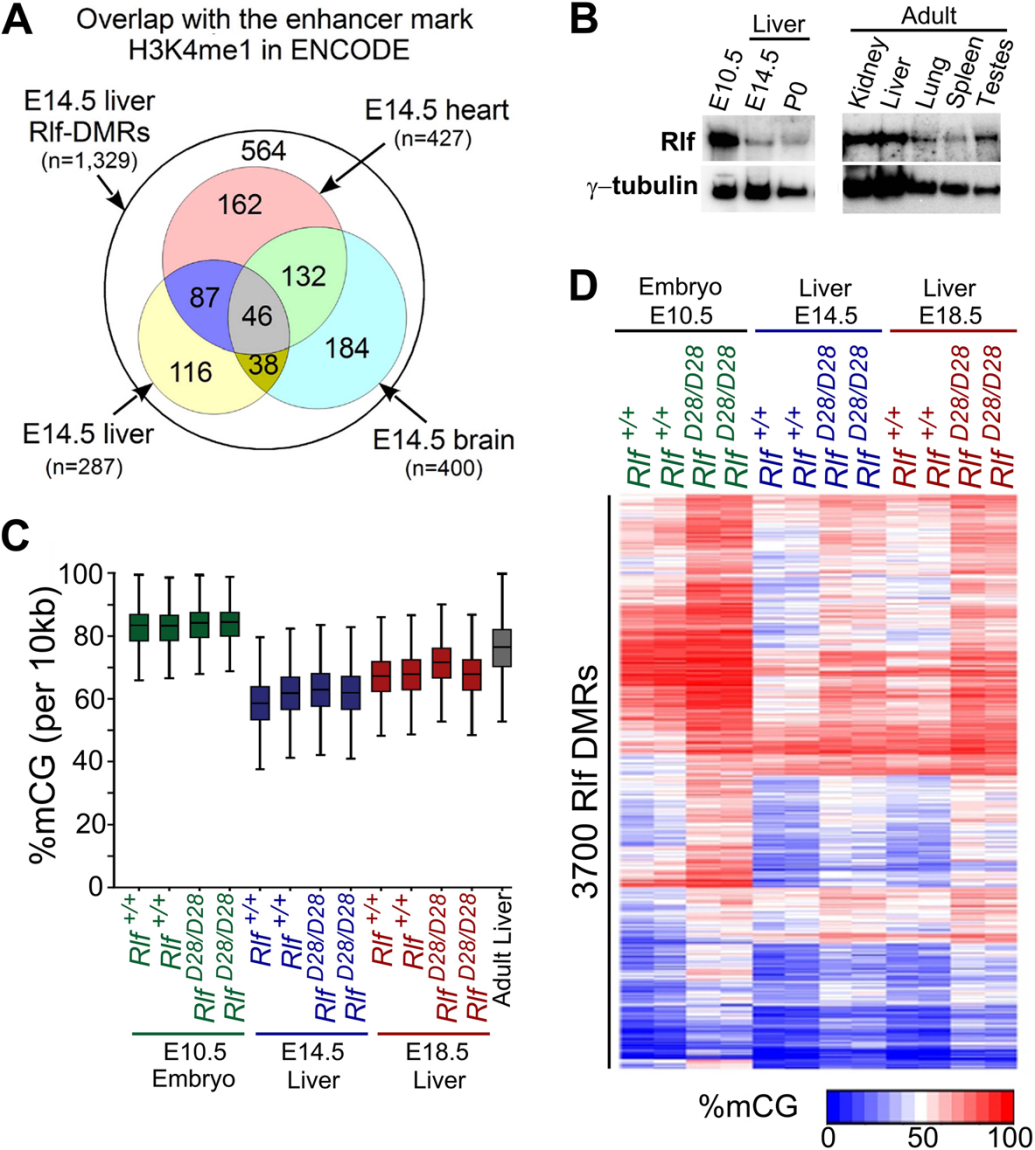
**Rlf-DMRs occur across a range of tissues and developmental stages. (A)** Overlap with the enhancer mark H3k4me1 in ENCODE E14.5 liver, heart and brain tissues. ChIP-seq enrichment was defined as elements with ChIP-seq RPKM greater than two-fold enriched over input RPKM for both replicates. **(B)** Representative Western blot showing Rlf protein expression in lysates prepared from fetal and adult wild-type mouse tissues. γ-tubulin was used as a loading control. **(C)** Box plots of percent mCG calculated from non-overlapping 10 kb bins for each whole genome bisulphite sequencing dataset used for this study. Additionally, the percent mCG distribution for adult liver (accession: GSM1051157); [5], obtained from the Gene Expression Omnibus [12], is included. The edges of the box-plot represent the 25th and 75th percentiles, the central bars indicate medians and whiskers indicate non-outlier extremes. **(D)** Hierarchical clustering showing methylation levels in E10.5 embryo, E14.5 liver and E18.5 liver for sites identified as Rlf-DMRs in E14.5 liver. Each row represents a Rlf-DMR and each data point represents the weighted average of the percent mCG for the Rlf-DMR in that sample.

Data from the ENCODE E14.5 liver showed that the majority of the E14.5 liver Rlf-DMRs (973 of the 1,329) were not enriched for H3K4me1, H3K27ac or H3K4me3, raising the possibility that many Rlf-DMRs might be active enhancers in another tissue or at a different stage of development. Indeed, others have noted differentially methylated regions of the adult genome that are not active enhancers but are likely to have been at an earlier stage in development, termed “vestigial” [5]. To test this idea, ENCODE’s H3K4me1 reads in E14.5 brain and heart were interrogated and an additional 478 E14.5 liver Rlf-DMRs were found to be enriched for H3K4me1 in one or other of these tissues (Figure 4A). This suggests that many, if not all, of the Rlf-DMRs without active histone marks in E14.5 liver were active as enhancers in other tissues.

Western blot analysis showed that Rlf is expressed in many adult, fetal and embryonic tissues (Figure 4B and data not shown). Based on this finding, we repeated the whole-genome bisulphite sequencing at both an earlier stage (E10.5 whole embryo) and a later stage (E18.5 liver). We used E18.5 liver because it has a different cell type make-up from E14.5 liver and the cell type ratios are known. At E14.5, the liver is the major haematopoietic organ, with 56% of cells being Ly76^+^ (erythroblasts) and 23% being Krt8^+^ Acta2^−^ epithelial cells (hepatoblasts). By E18.5, erythroblasts represent only 7% of the total cells and hepatoblasts become the dominant cell type, making up 82% of the total cell population [13]. We collected two wild-type and two homozygous samples in each case and sequenced to 15 fold depth. In order to make comparisons between these datasets and our previous datasets (E14.5 liver), we carried out subsampling to equalise the depth of reads across all three (see Methods). Interrogation of a random selection of 1 kb fragments across the genome confirmed that subsampling had little effect on the weighted average of methylation in these regions (Additional file 5: Figure S3). The global CpG methylation level, shown as CpG methylation levels averaged across 10 kb windows, differed across the three tissue types but in each case the overall level was unaffected by lack of Rlf (Figure 4C).

A total of 1,199 Rlf-DMRs were identified from the E14.5 liver dataset (Additional file 6: Table S3). The discrepancy between this figure and the 1,329 previously identified is due to the subsampling and slightly altered parameters used (see Methods). Additionally, 2,206 were called from the E10.5 whole embryo dataset and 1,514 from the E18.5 liver dataset (Additional file 6: Table S3), resulting in a total of 4,919 Rlf-DMRs across all three datasets.

After merging overlaps, 3,700 remained as unique coordinates. Many were shared and, as expected, the majority were more methylated in the mutants. From the heat-plot it is evident that the Rlf-DMRs showed surprisingly similar patterns of methylation change in the two liver datasets, despite quite different cell type populations (Figure 4D).

We then asked how many of these 3,700 were differentially methylated in one or two but not all of the three datasets. 986 were found to have a smaller than 5% methylation difference in at least one tissue, suggesting that they were not Rlf-DMRs in that tissue (Additional file 7: Figure S4).

Because of the large change in cell type between the E14.5 liver and E18.5 liver, we were interested to see how many Rlf-DMRs were different across these two datasets. We found only 200 Rlf-DMRs that were called in one but not the other (Additional file 8: Figure S5).

In summary, Rlf-DMRs were identified in all tissues/stages tested and a subset was unique to each dataset.

Gene ontology analysis of the genes closest to the 3,700 Rlf-DMRs failed to identify any specific class of gene except for an enrichment for genes encoding transcription factors (data not shown). Because Rlf-DMRs were found at regulatory elements, we searched for enriched consensus DNA binding motifs at Rlf-DMRs. No motifs were found to be significantly enriched over background (data not shown).

### Rlf interacts with chromatin-associated proteins involved in transcription and replication and binds DNA *in vitro*

**Figure 5:**
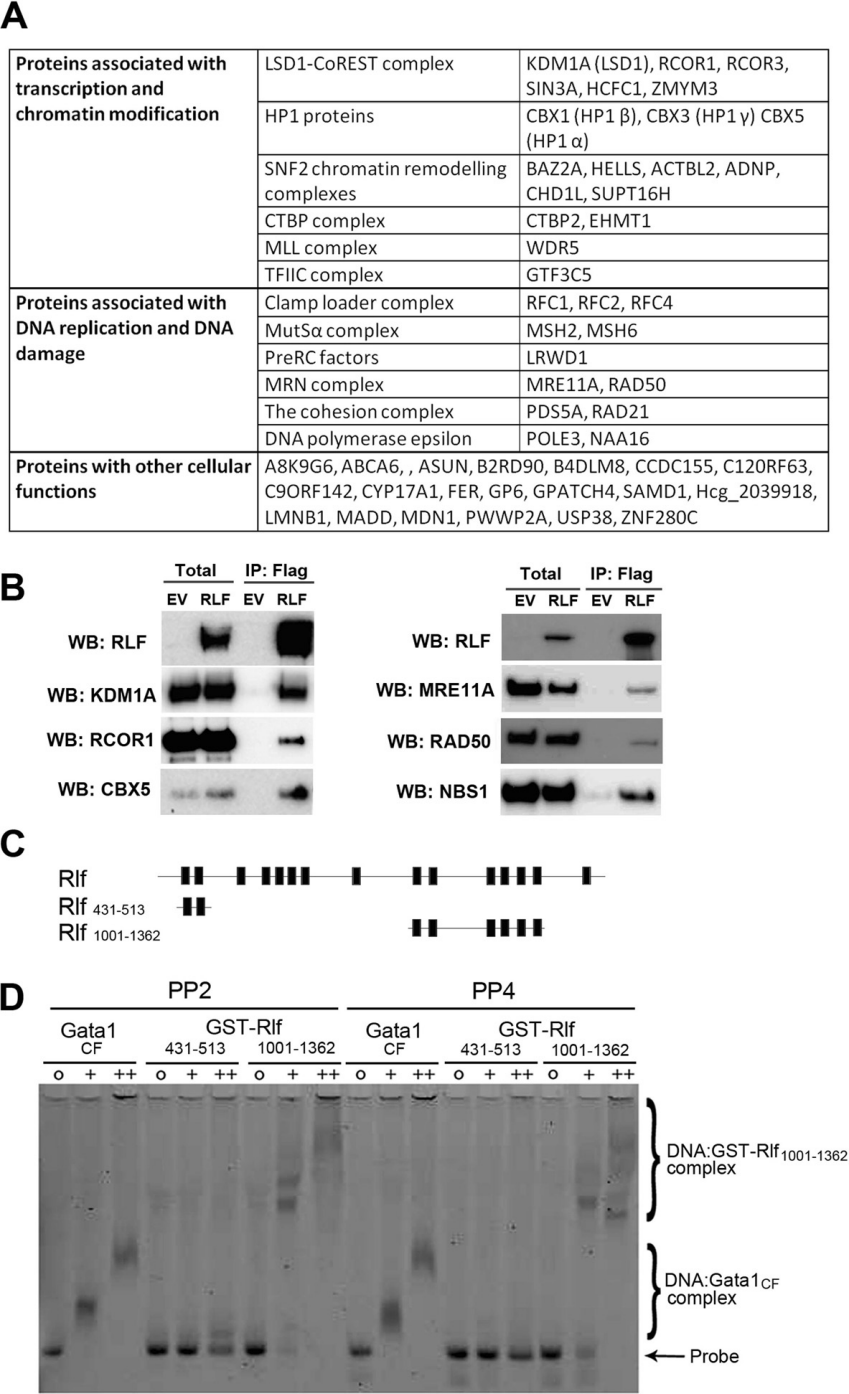
**Rlf interacts with DNA and with proteins associated with transcription, chromatin modification and DNA replication and/or repair. (A)** Summary of RLF interacting proteins determined by co-IP and mass spectrometry of HEK293T cells expressing RLF-Flag or an EV control (for details, see Additional file 9: Table S4). Proteins are grouped into categories and complexes based on previously reported functions. Three independent biological replicates were analysed per condition. Proteins classified as interacting partners were significantly enriched in RLF-Flag samples, *p* < 0.05, and are supported by at least five peptides. **(B)** Western blots showing co-IP of endogenous KDM1A, RCOR1, CBX5, MRE11A, RAD50 and NBS1 with exogenous RLF-Flag in transiently transfected HEK239T cells. Input represents 1% of the nuclear extract used for immunoprecipitation. Representative Westerns from at least three independent experiments are shown. **(C)** Schematic representation of mouse Rlf showing putative zinc finger domains. GST-fusion proteins used in EMSA experiments, Rlf_431–513_ and Rlf_1001–1362_, are also presented. **(D)** EMSA analysis testing the capability of GST-RLF fusions, Rlf_431–513_ and Rlf_1001–1362_, to bind to FAM labeled oligonucleotide probes, PP2 (lanes 4–9) or PP4 (lanes 13–18). Gata1_CF_, which is known to interact with DNA, was used as a positive control (lanes 1–3 and 10–12).

Given that Rlf was found to be required for normal DNA methylation status at regulatory elements throughout the genome, we were interested to find protein partners. Co-immunoprecipitation (co-IP) experiments were performed. Human HEK293T cells expressing either a Flag-tagged RLF cDNA (RLF-Flag) or an empty vector control (EV) were generated via transient transfection. Anti-Flag conjugated magnetic beads were used to co-IP tagged RLF from nuclear lysates. Eluted proteins were identified by mass spectrometry. Three biological replicates were used in each case and the data were analysed using Scaffold (see Methods). Interactions were considered significant if they showed at least a 5-fold enrichment in RLF-Flag samples compared to EV samples. A complete list of binding partners is shown in Figure 5A and the number of peptides detected in each biological sample is shown in Additional file 9: Table S4. Many of the identified proteins have established roles in transcription and chromatin modification, or in replication and DNA repair, as indicated (Figure 4A). A subset of the binding partners was tested using Western blot analysis of independent samples (Figure 5B). All that were tested in this way, validated.

To test whether Rlf has the capacity to bind DNA, we performed electrophoretic mobility shift assays (EMSAs) using two non-overlapping sub-fragments of Rlf, Rlf_431–513_ and Rlf_1001–1362_, that contain two and six zinc fingers, respectively (Figure 5C & D). Recombinant GST-fusion Rlf protein fragments were purified from *E. coli* and incubated with Fluorescein amidite (FAM)-labelled oligonucleotide probes, called pentaprobes, which have been used previously to test DNA binding [14]. A protein fragment containing the C-terminal zinc finger of Gata1, which is known to bind DNA, was included as a positive control. Incubation with either Rlf_1001–1362_ or Gata1_CF_ resulted in a shift in the migration pattern of both probes. No shift was detected with Rlf_431–513_ fragment (Figure 5D). The results suggest that Rlf binds DNA directly, but not all zinc fingers in Rlf mediate DNA interactions.

### Loss of Rlf affects mRNA levels of some genes in E14.5 fetal liver

**Figure 6:**
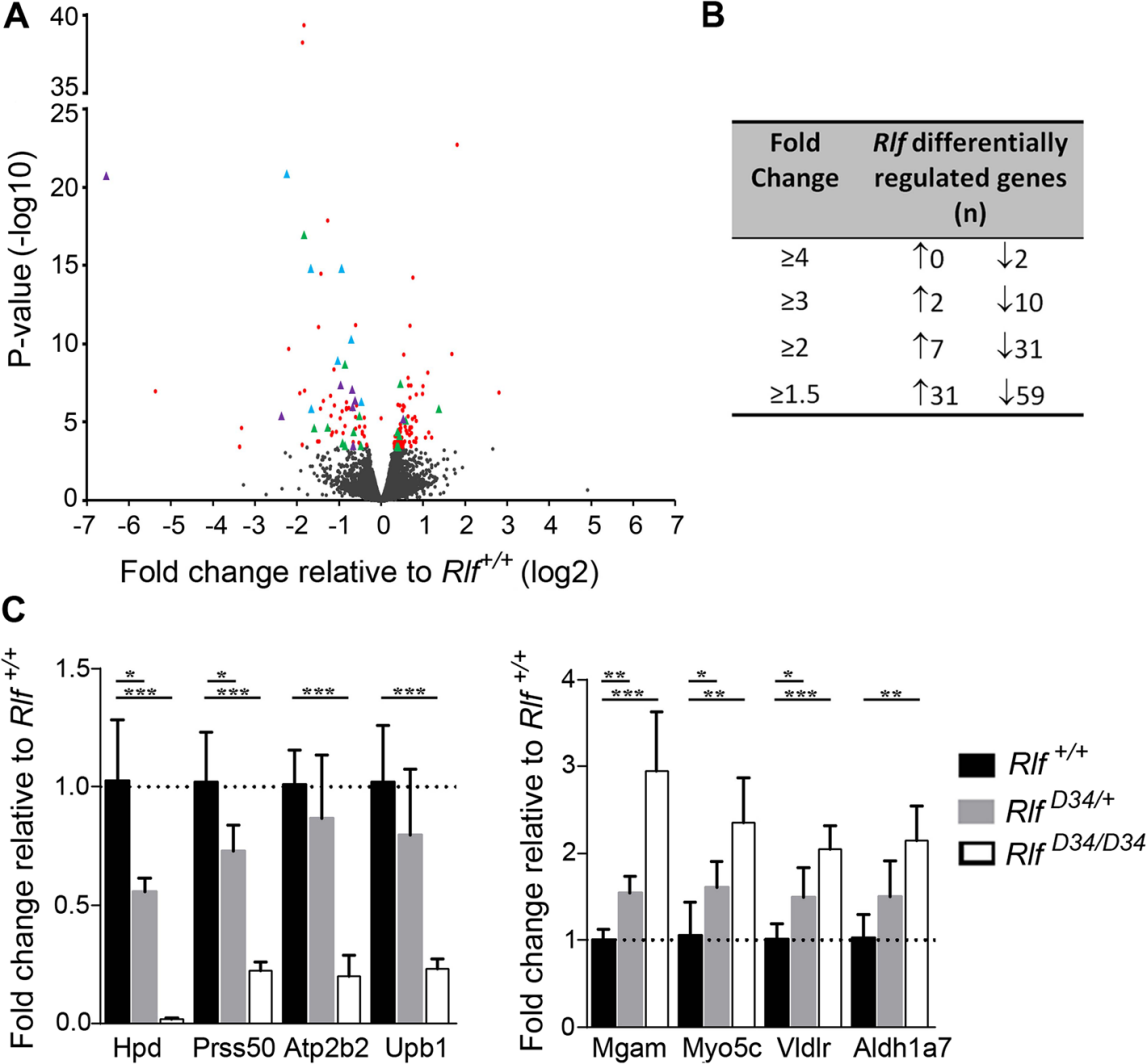
**Loss of Rlf influences transcription in the fetal liver and is associated with alterations in epigenetic state. (A)** Volcano plot depicting the results of RNA-seq analysis of RNA from E14.5 livers of *Rlf* ^*+/+*^and *Rlf* ^*MommeD28/MommeD28*^ mice. Three independent biological replicates were analysed per genotype. Significantly differentially expressed genes are presented as red, blue, green or purple data points, *p* < 0.05. Genes that have either a Rlf-DMR or a Rlf-dependent change in H3K4me1 occupancy within 50 kb of their TSS are highlighted by green and purple triangles, respectively. Blue triangles highlight genes with a change in both DNA methylation and H3K4me1 occupancy. Black data points represent genes whose expression was not significantly altered by loss of *Rlf.* **(** ***B*** **)** Table showing the number of genes significantly differentially expressed in *Rlf* ^*MommD28/MommeD28*^ E14.5 livers relative to *Rlf* ^*+/+*^ controls at various fold-change cut-offs (*p* < 0.05). ↑ and ↓ indicates increased or reduced expression, respectively, in *Rlf* ^*MommeD28/MommeD28*^ homozygotes. **(C)** Quantitative real-time RT-PCR analysis of putative Rlf-regulated genes identified via RNA-seq. Analyses used RNA extracted from E14.5 fetal livers of wild-type mice and an independent null *Rlf* mutant mouse line*, MommeD34,* see Introduction*.* Mean ± SEM is present for at least six individuals per genotype. Statistical significance was determined via a t-test *p < 0.05, **p < 0.005.

To address whether the absence of Rlf influenced transcription, we conducted RNA-seq on polyadenylated RNA from E14.5 fetal liver of wild-type and *Rlf* homozygous mutants. A relatively small number of significant changes were detected, using an adjusted *p*-value <0.05 (Figure 6A, red circles and green, blue and purple triangles, and Additional file 10: Table S5). A similar number of up and down-regulated genes were observed relative to wild-type controls. We asked if these differentially expressed genes were proximal to either a Rlf-DMR (green triangle) or a site with reduced H3K4me1 occupancy (purple triangle) or both (blue triangle) in the mutant. These were identified and were found to preferentially partition to the genes with reduced expression in the mutants, consistent with an association with transcription. Most of those with a greater than 1.5-fold change showed reduced expression in the mutants (Figure 6B).

Ingenuity pathway analysis of significantly differentially expressed genes (*p* < 0.05) showed an over-representation of genes involved in the following networks: Cardiovascular System Development and Function, Organismal Development, Tissue Development (*p* score = 52); Cancer, Cellular Development, Cellular Growth and Proliferation (*p* score = 30); and Lipid Metabolism, Small Molecule Biochemistry, Nucleic Acid Metabolism (*p* score = 19). Real-time RT-PCR using RNA from an independent *Rlf* mutant mouse line, *Rlf*^*MommeD34/MommeD34*^, was used to validate RNA-seq findings for four up-regulated and four down-regulated genes (Figure 6C). In several cases, heterozygosity for the *Rlf* mutant allele also resulted in significant changes in gene expression (Figure 6C), suggesting that dosage of Rlf might be critical in some contexts.

Taken together with our earlier findings, this data supports a role for Rlf in transcription.

## Discussion

Cytosine methylation has a fundamental role in differentiation and development in mammals but the mechanism is unclear. Three recent studies of differential methylation in mice and humans found hundreds of thousands of regions that were differentially methylated across different tissues, called ts-DMRs [4,5,7]. Many of these differentially methylated regions overlapped with transcription factor binding sites and with histone marks associated with active enhancers (H3K4me1 and H3K27ac). Here we report that the recently identified epigenetic modifier, Rlf, is involved in the process of establishing and/or maintaining low levels of DNA methylation at a subset of these *cis*-regulatory elements. The enhancers involved are not specific to one or a few cell types nor can they be assigned to genes of one of a few functional categories. Our studies, carried out in only three different tissues/stages in development have revealed 3,700 unique locations but studies in additional cell types are likely to reveal many more.

One of the interesting observations made in the studies identifying ts-DMRs was that many lacked the H3K4me1 and H3K27ac marks associated with active or poised enhancers, even in the tissues in which the locus was hypomethylated, raising the possibility that the regions had been active early in development, had become inactive but remained hypomethylated. The term “vestigial enhancer” was used to describe this class [5]. Our results show that Rlf is also involved in the maintenance of hypomethylation at such sites.

Independent studies have shown that most tissue-specific differential methylation occurs at CpG island shores [15] and others have speculated that the methylation of these sites will turn out to be transcription factor dependent [16]. Our findings support this model, with the absence of Rlf resulting in increased DNA methylation at CpG island shores.

Mice null for *Rlf* weigh noticeably less at late gestation and die shortly after birth [1]. Here we report widespread effects on DNA methylation that are observed as early as mid-gestation (E10.5). RNA-seq on E14.5 embryonic livers reveals that a number of genes were affected by the absence of Rlf, although these effects are relatively subtle. The majority of these genes are down regulated in homozygous *Rlf* mutants. Many of these changes are associated with the expected chromatin modifications i.e. increased DNA methylation and decreased H3K4me1. Taken together this suggests a role for Rlf in transcriptional activation. The relatively mild nature of the transcriptional changes observed might indicate that in some settings the loss of Rlf alone is not sufficient to alter transcription. Further study is required to uncover the precise mechanism through which Rlf influences gene expression.

Electrophoretic mobility shift assays indicate that Rlf can interact with DNA directly and it has been proposed that the binding of transcription factors, such as CTCF and REST, at transcriptional regulatory regions is sufficient for hypomethylation [6]. These binding events could act simply by preventing the binding of other factors such as DNA methyltransferases or by actively recruiting factors such as Tet enzymes. The co-immunoprecipitation studies support the idea that Rlf acts in concert with other proteins that have known roles in transcriptional regulation. The histone demethylase, KDM1A, which co-immunoprecipitated with RLF, is normally associated with transcriptional silencing. However, it has also been found in a complex with the androgen receptor and in this context it acts as an activator by demethylating H3K9me2 marks at transcriptional enhancers [17]. This mechanism would be consistent with the predominantly activating effect of Rlf on transcription.

Although ChIP-seq studies are likely to shed light on these possible mechanisms in terms of genome occupancy and co-occupancy, suitable antibodies for Rlf are not currently available. In the absence of ChIP-seq data for Rlf, we have used whole genome bisulphite sequencing, ChIP-seq for H3K4me1 and RNA-seq as tools to identify regions of the genome where the absence of Rlf has an effect, linking it to enhancers and transcriptional activation. We have also found that the global levels of CpG methylation are not changed by lack of Rlf, making indirect mechanisms e.g. via changes to DNA methyltransferase activity, an unlikely explanation for our findings.

Whereas proteins with closely spaced arrays of zinc fingers (such as Krϋppel zinc finger proteins) are relatively common in the genome and are well known as DNA-binding factors, proteins with widely-spaced zinc fingers, like Rlf, are less common and are poorly characterised. The significance of the wide spacing is unknown [18]. Interestingly, several other widely-spaced zinc finger proteins have been identified in association with epigenetic programming. For example, Su(var)3-7 was identified in the original *Drosophila* screen for modifiers of position effect variegation [19], and Wiz, was identified in our screen for modifiers of epigenetic reprogramming in mice [1]. Only a small number of additional widely-spaced zinc finger proteins have been described in the mouse, including the Rlf paralog Zfp292 [20] and Peg3 [21], both of which can bind DNA, and Zfp346 (Jaz) and Zmat3 (Wig-1), both of which can bind double-stranded RNA [22,23]. Despite being derived from the same ancestral gene, Rlf and Zfp292 have diverged considerably, including at the putative zinc fingers. Further studies will reveal how similar these proteins are in their functions.

## Conclusion

Overall our data show that Rlf has a prominent role in the modulation of epigenetic state at *cis*-acting regulatory regions across the genome. Future studies of this protein will reveal the mechanisms that underlie this process.

## Methods

### Mouse lines

*MommeD28* and *MommeD34* mouse lines were produced in an ENU mutagenesis screen for epigenetic modifiers and have been described previously [1]. The ENU screen was carried out in *Line3* FVB/NJ mice carrying a multi-copy GFP transgene under the control of the human alpha-globin promoter and the HS-40 enhancer. All mice used in this study were homozygous for the GFP transgene array. ENU mutant lines have been maintained as inbred colonies by backcrossing to unmutagenised *Line3* mice for at least ten generations. All procedures were carried out with approval from the Animal Ethics Committee of the QIMR Berghofer Medical Research Institute.

### DNA and RNA extraction

Embryonic tissues were obtained from natural timed-matings of heterozygous individuals, with the presence of a post-coital plug defined as E0.5. Pregnant dams were euthanised via cervical dislocation at the required time point. Whole embryos (E10.5) or fetal livers (E14.5 & E18.5) were removed and homogenised in RLT Plus Buffer (Qiagen, Doncaster, VIC, Australia) using a syringe and needle. Genomic DNA and total RNA were prepared using an AllPrep DNA/RNA Mini Kit according to manufacturers’ instructions (Qiagen, Doncaster, VIC, Australia). Genotyping of DNA was performed by Sanger sequencing. Primers: *MommeD28* Geno F: TCT GCC AGT CTC TGA AGA AAA GGC A, *MommeD28* Geno R: CTG TGG CTT TCA TTC TGG AAG GGA A; *MommeD34* Geno F: GTTGCCCATTTTAGGGGAG, *MommeD34* Geno R: TGGATTGCACCCTGGACTAC.

### Whole Genome Bisulphite Sequencing

Whole Genome Bisulphite Sequencing was performed by the Centro Nacional de Análisis Genomico (CNAG, Barcelona, Spain). Genomic DNA samples were spiked with λ DNA and sheared by sonication. Libraries were prepared using the TruSeq Sample Prep Kit (Illumina, San Diego, CA) and underwent two rounds of sodium bisulphite conversion using the EpiTect Bisulphite Kit (Qiagen, Doncaster, VIC, Australia). 100 bp paired end sequencing was performed on the Illumina HiSeq 2000. Sequencing reads were trimmed for poor quality at their 3′ ends using the program trim_seq [24] with the options -l 3 -m 3. Reads were mapped to mouse genome (NCBI37/mm9) after first converting known FVB SNPs to the FVB state using the tool FastaAlternate from the GATK package [25]. The SNP positions were obtained from Sanger (rsIDdbSNPv137) and coordinates converted from the NCBI38/mm10 to NCBI37/mm9 using the LiftOver tool from UCSC [26]. Only nucleotide substitutions were used to maintain genome length. The lambda genome sequence and the sequence for the GFP transgene [27] were added to the genome sequence as separate chromosomes. Sequencing reads were mapped to the new genome using the program Bismark, version 0.7.12, [28] combined with Bowtie2, version 2–2.1.0 [29], using the options -N 1 -D 30. The resulting files were filtered for PCR duplicates using the program deduplicate_bismark_alignment_output.pl and CpG methylation values were then extracted using the program bismark_methylation_extractor with the options --ignore_r2 2 --counts --bedGraph --paired-end --no_overlap --comprehensive --merge_non_CpG [28]. The CpG methylation calls (one for each strand) were merged to a single methylation call for each CpG using custom scripts.

For the full E14.5 liver datasets, differentially methylated regions were identified using the R package bsseq [30]. The smoothing was carried out with the options ns = 20, h = 250, maxGap = 100,000,000. Loci with coverage ≥8 in at least one biological replicate for wild-type and mutant were retained and Rlf-DMRs were identified using t-statistic quantile cut-offs of 0.01 and 0.99, requiring >10 CpGs per Rlf-DMR and a mean methylation change >15%.

The 10.5 embryo dataset, the E18.5 liver dataset and the sub-sampled E14.5 liver dataset (sub-sampled to equal the mean library read-counts of the 10.5 embryo and the E18.5 liver datasets) were mapped to the genome and methylation values obtained, as described above. To enable comparison between the tissues, methylation values for CpGs covered by at least 6 CpGs in all samples were extracted and Rlf-DMRs called across mutants and wild-types for each tissue using bsseq, smoothing with the options ns = 50, h = 500, maxGap = 10,000,000, requiring a coverage of at least 6 in both replicates and a maximum gap of 1,500 bp between CpGs in a Rlf-DMR. Rlf-DMRs with ≥8 CpGs and mean methylation change >15% were used for downstream analyses.

Methylation values used for hierarchical clustering were the weighted averages of CpG methylation within each genomic coordinate. Only coordinates containing at least 8 CpGs with coverage of at least 6 reads across all samples, were used.

### Enrichment of histone marks

To calculate enrichment of histones at Rlf-DMRs the ENCODE sequencing reads [31] for both biological replicates, and for the input, for each tissue and histone modification considered in this manuscript (E14.5 liver, E14.5 heart and E14.5 brain), were obtained from the UCSC Genome Browser [32]. Datasets with longer reads were 3′ trimmed such that all datasets contained reads of equal length. The reads were subsequently mapped to the mouse reference genome (NCBI37/mm9) using the program Bowtie2 with the options --trim5 6 --local -L 22 -N 1 retaining only uniquely mapped reads. Reads likely to be PCR duplicates were identified and removed using the program MarkDuplicates Picard [33]. Enrichment of histone modifications were calculated as described in [5], requiring >2-fold enrichment over input for both replicates.

### Gene ontology analyses, DNA sequence motif search and conservation scores

Gene ontology analyses of genes proximal to Rlf-DMRs were carried out using the GREAT tool [34]. The Homer tool, version 4.6 [35], was used to investigate for enrichment of known and novel transcription factor binding motifs at Rlf-DMRs using the options -size given –cpg, and otherwise default parameters. PhastCons conservation scores [36] for placental mammals, obtained from the UCSC Genome Browser [37], were used to get average conservation scores for Rlf-DMRs.

### Bisulphite sequencing at Smad 3–6 locus

Bisulphite conversion of genomic DNA was carried out using the EpiTect Bisulphite Kit (Qiagen, Doncaster, VIC, Australia). Nested PCRs were performed to interrogate methylation at the Smad3-6 intergenic region with the following primers: Smad3-6 bis F1: AAGTGGAATTTTTTAGTGGTAGATG; Smad3-6 bis R1: AACTACTTTAATAAAAAATAACATAACC, Smad3-6 bis F2: TTGGTATGTGTTGTTTTTAGTTTTG and Smad3-6 bis R2: ACAATTTAACTATTCATTATATCTCTAACA. Cycling conditions were as follows: Primary PCR, 94°C for 2 minutes for 1 cycle; 94°C for 30 secs, 53°C for 30 secs, 72°C for 45 secs for 35 cycles and 72°C for 6 mins for 1 cycle. The secondary PCR was performed using an annealing temperature of 51°C*.* The PCR product was ligated into pGEM T Easy (Promega, Annandale, Australia) and transformed. DNA from individual colonies was sequenced using Sanger sequencing. The bisulphite conversion rate was ≥98% and sequences were analysed using BiQ Analyser software [38].

### H3K4me1 ChIP-seq

H3K4me1 ChIP-seq library preparation, sequencing and initial bioinformatical analyses were carried out by ActiveMotif. Briefly, the prepared libraries were sequenced on the Illumina NextSeq 500 platform using a read length of 75 nt. Each resulting dataset was sub-sampled to the read-count of the library with the smallest number of reads and aligned to the mouse genome (mm9) using BWA [39] with default parameters, allowing a maximum of two mismatches. Only uniquely mapping reads were retained and reads identified as PCR duplicates were discarded.

Mapped reads were then extended from their 3′ ends to a length of 200 bp to account for the length of the ChIP fragments used to prepare the libraries. The density of fragments along the genome was obtained by dividing the genome into 32-nt bins and the number of fragments overlapping each bin was determined.

Regions enriched for H3K4me1 (i.e. peaks) were determined using the program MACS [40] using default parameters, and the resulting peaks from the four datasets merged into a final set (union of peaks), which was then used for the presented analyses.

The density of H3K4me1 reads across the GFP transgene was determined by mapping 10,000,000 reads from each library to a mouse genome (mm9) with the sequence of the transgene added as an additional chromosome. The reads were mapped using the program Bowtie2 [29] using default parameters and retaining those reads with a minimum mapping quality of 40.

### co-IP/Mass Spec

HEK293T cells (ECACC, Salisbury, UK) were cultured in Dulbecco’s Modified Eagle Medium (DMEM) with 10% fetal bovine serum (FBS) (Life Technologies, Mulgrave, VIC, Australia). Cells were transfected with a pCMV6-Entry vector containing Myc-DDK-tagged-Human rearranged L-myc fusion, RLF-Flag or vector alone, EV (Rockville, MD, USA) using Lipofectamine 2000 (Life Technologies, Mulgrave, VIC, Australia). After 48 hrs, the cells were lysed with a hypotonic lysis buffer, nuclei were isolated and resuspended in digestion buffer with PMSF and protease inhibitors (Active Motif, Carlsbad, CA, USA). For each replicate, 4 mg of nuclear lysate was immunoprecipitated using anti-FLAG M2 magnetic beads (Sigma-Aldrich, St. Louis, MO, USA). IP reactions were carried out in High stringency IP buffer supplemented with protease inhibitor cocktail, for 1 hr at 4°C. Samples were eluted with 8 M Urea. Digestion and IP buffers were from the Nuclear Complex Co-IP kit (Active Motif, Carlsbad, CA, USA). Eluted proteins were analysed via mass spectrometry at the La Trobe University Mass Spectrometry Facility. Samples were digested with 1 μg of trypsin overnight at 37°C and peptide fragments analysed via tandem mass spectrometry on an Orbitrap Elite mass spectrometer (Thermo Scientific). Three biological replicates were analysed per condition, with three replicate injections performed for each. MS/MS spectra were queried using Thermo Scientific Proteome Discoverer software v. 1.4 with Mascot (v. 2.4.0 Matrix Science) search engine. Result files were imported into Scaffold (Proteome Software Inc.) for visualisation and validation. Putative interacting proteins were considered significant if they showed at least a five-fold enrichment in RLF-Flag samples compared to EV, with a *p*-value of <0.05, with at least five peptides detected.

### Western Blotting

Protein lysates from whole tissues were prepared by homogenising in ten volumes of urea lysis buffer, as described [1]. Samples were quantified using a BCA assay (Thermo Scientific, VIC, Australia), separated on polyacrylamide gels (Bio-Rad, Gladesville, NSW, Australia) and immunoblotted. Clarity Western ECL substrate was used for visualisation (Bio-Rad, Gladesville, NSW, Australia). Antibodies used were as follows: anti-RLF, Ab115011 and anti-RCOR1, Ab32631 (Abcam, Cambridge, UK); anti-LSD1, 2139 and anti-HP1α, 2616 (Cell Signaling, Danvers, MA, USA); anti-Flag M2, F3165 and anti-γ-tubulin, T5192 (Sigma-Aldrich, St. Louis, MO, USA); anti-MRE11 (12D7) GTX70212, anti-NBS1 (1C3) GTX70222 and anti-RAD50 (13B3) GTX70228 (GeneTex, Irvine, CA).

### Protein production and purification

Rlf sub-fragments were amplified from mouse cDNA using the following primers, Rlf_431_ F: GCGGATCCGGTTCCTCTGAGAGATACCAGAG, Rlf_513_ R: GCGAATTCCTATTAAGCGCTTTTCAATAGTAATTTCTT, Rlf_1001_ F: GCGGATCCAGTCAGTACCTTGCACAGTTGGC, Rlf_1362_ R: GCGAATTCCTATTA GCTGCTGAACAGGTTGTCATAATA and cloned into pGEX-6P, for expression with an N-terminal GST tag, using BamHI and EcoRI restriction sites. GST-Rlf protein sub-fragments were overexpressed in *Escherichia coli* Rosetta II cells with induction by IPTG. Cells were lysed by sonication in lysis buffer (50 mM MES, pH 6.5, 1 M NaCl, 1.4 mM β-mercaptoethanol, 1 mM PMSF), incubated with 1 mg/mL DNase for 30 mins at 4°C, and further sonicated. The soluble fraction was loaded onto glutathione-sepharose resin and washed (50 mM MES, pH 6.5, 1 M NaCl, 10% (v/v) glycerol, 1.4 mM β-mercaptoethanol, 1 mM PMSF). GST-Rlf recombinant protein sub-fragments were eluted (50 mM MES, pH 6.5, 150 mM NaCl, 50 mM reduced glutathione, 1.4 mM β-mercaptoethanol, 1 mM PMSF) and further purified by size exclusion chromatography on a HiLoadTM Superdex 75 column (GE Healthcare Life Sciences, Little Chalfont, United Kingdom).

### EMSAs

EMSAs were performed using FAM labeled pentaprobe oligonucleotides (Integrated DNA Technologies, Coralville, IA, USA). PP2: FAM-TATCTTACTTTAGTTTCATTTAATTGT GTTGTACTCTCCTCTGCGTTCACTTAGCTTAACTTGGTTTGGCTTGATTTGACTTCAGTTGCGCTCTATTCTA; PP4: FAM-TATCCTACCCATTGGGCTCATCTGATCCATCCGGTCCCGTCCACTCGGCTATGTTATGCTGTATTGCAGTCGTGTCGCGTCGAGCTGCCCTAATCCCACC. Reactions were performed in MSB buffer (10 mM HEPES, pH 7.9, 30 mM NaCl, 1 mM MgCl_2_, 20 μM EDTA) containing 10 mM DTT, 10 μM ZnSO_4_, 67 μg/mL acetylated bovine serum albumin and 4% Ficoll. All reactions contained 5 nM oligonucleotide and either 0.5 μM, 5 μM or no protein. Samples were incubated on ice for 30 min, separated on non-denaturing polyacrylamide TBE gels and visualised using a Typhoon FLA 9500 scanner (GE Healthcare Life Sciences, Little Chalfont, United Kingdom).

### RNA-seq

mRNA-seq library preparation (Illumina TruSeq RNA Sample Preparation kit; Illumina, San Diego, CA) and Illumina HiSeq 2000 Sequencing with 50 bp single-end reads was performed by the Australian Genome Research Facility (AGRF, Melbourne, VIC, Australia). Reads were aligned to the mouse genome (NCBI37/mm9) using Tophat [41] with the parameters -I 100000 --no-coverage-search --read-mismatches 2 --library-type fr-unstranded. Read counts for mRNA transcripts were extracted from the mapped reads using htseq-count [42] with the options -s no -m intersection-strict and using gene annotations from Ensembl ([43], release 67). Differential gene expression was assessed using the R-package DESeq, using default parameters [44].

### Quantitative real-time RT-PCR

RNA was reverse transcribed using iScript Reverse Transcription Supermix (Bio-Rad, Gladesville, NSW, Australia). Quantitative real-time reverse transcription (RT)-PCR analysis was performed on a Corbett Research Rotor-Gene (Qiagen, Doncaster, VIC, Australia) using Platinum SYBR Green qPCR Supermix-UDG (Life Technologies, Mulgrave, VIC, Australia). Expression of β-actin was used for normalisation. Statistical analysis was performed using Student’s *t* test. Primer sequences used are as follows: Aldh1a7 F: GCAGGGAAAAGCAATCTGAA, R: TCTGACCCTGGTGGAAGAAC; Vldlr F: TCGGGCTTTGTTTACTGGTC, R: AGTAGAGGCGGCTTTTGACA; Mgam F: TTGTTCTGCTGCTTGTCCTG R: ACTGGGCAATTGGGAGAGTT; Myo5c F: GGCTGAAATCGCAAAGGACT R: CTCATGGAGGTAGCTGAGGG; Hpd: F: AGGTAGTCAGCCACGTCATC, R: CAATGTGGTCGCAGTCCAGC; Prss50: F: GGTTCATTCCAGCAACCTCC, R: GAAGCGATAAGGATGCCAGC; Atp2b2 F: CCTCAAAACCTCGCCTGTTG, R: GTGGGTGGTAGAAGGACAGT; Upb1 F: AGGAATCTCGATCTGCCCAG, R: ATTGACTCCACACATTGCGG.

### Availability of supporting data

All methylome and RNA-seq datasets used in this study have been submitted to the NCBI Gene Omnibus (GEO; http://www.ncbi.nlm.nih.gov/geo/) under accession number GSE58108.

## Additional files

Additional file 1: Figure S1. Read coverage at CpG dinucleotides across the mouse genome. Additional file 2: Table S1. E14.5 Rlf-DMRs identified using genome-wide bisulphite sequencing of DNA from fetal livers of *Rlf+/+* and *RlfMommeD28/MommeD28* mice. Additional file 3: Figure S2. Hierarchical clustering of E14.5 Rlf-DMRs. Additional file 4: Table S2. H3K4me1 peaks detected in chromatin extracted from *Rlf* ^*+/+*^ and Rlf^*MommeD28/MommeD28*^ E14.5 fetal livers. Additional file 5: Figure S3. Hierarchical clustering of %mCG for random 1 kb regions from the bisulphite converted genomes prepared for this study. Additional file 6: Table S3. E10.5 Embryo, E14.5 liver (down-sampled) and E18.5 liver Rlf-DMRs identified using genome-wide bisulphite sequencing of DNA from *Rlf* ^*+/+*^ and *Rlf* ^*MommeD28/MommeD28*^ whole embryos or fetal liver. Additional file 7: Figure S4. Hierarchical clustering of Rlf-DMRs with normal methylation in at least one tissue. Additional file 8: Figure S5. Hierarchical clustering of Rlf-DMRs detected for only one liver timepoint. Additional file 9: Table S4. Rlf interacting proteins identified via co-IP of HEK293T nuclear lysates, expressing RLF-Flag or an empty vector, with anti-Flag magnetic beads. Columns indicate the number of supporting peptides for each protein identified using Mass Spec. Additional file 10: Table S5. Differential expression analysis comparing RNA-seq datasets from *Rlf* ^*+/+*^ mice to *Rlf* ^*MommeD28/MommeD28*^ mice.

## Abbreviations

bp: Base pair
DMR: Differentially methylated region
ENU: N-ethyl-N-nitrosourea
GFP: Green fluorescent protein
H3K4: Histone H3 lysine
Momme: Modifier of murine metastable epiallele
PCR: Polymerase chain reaction
Rlf: Rearranged L-Myc Fusion
RPKM: reads per kilobase per million
