# Supplementary material for: The recently identified modifier of murine metastable epialleles, Rearranged L-Myc Fusion, is involved in maintaining epigenetic marks at CpG island shores and enhancers
Source: BMC Biol. 2015 Mar 26;13:21. doi: 10.1186/s12915-015-0128-2 (PMC4381397; doi:10.1186/s12915-015-0128-2)
Supplement: Additional file 1: Figure S1. — Read coverage at CpG dinucleotides across the mouse genome. [file 12915_2015_128_MOESM1_ESM.pdf]

Supplemental Figure 1

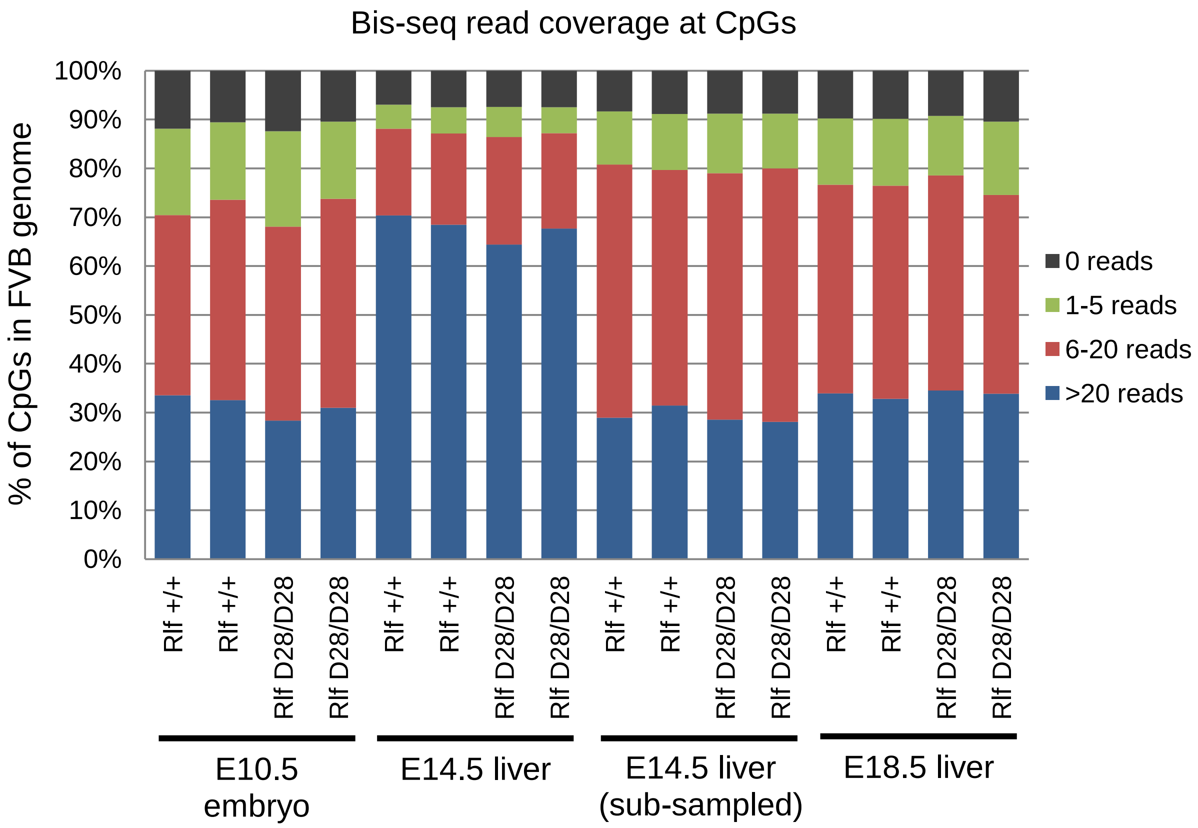

Read coverage at CpG dinucleotides across the mouse genome

For each CpG in the genome (n=21,729,695 after converting FVB SNPs), the read coverage was calculated and is summarized for each dataset in the bar-chart above.
