# Supplementary material for: The recently identified modifier of murine metastable epialleles, Rearranged L-Myc Fusion, is involved in maintaining epigenetic marks at CpG island shores and enhancers
Source: BMC Biol. 2015 Mar 26;13:21. doi: 10.1186/s12915-015-0128-2 (PMC4381397; doi:10.1186/s12915-015-0128-2)
Supplement: Additional file 3: Figure S2. — Hierarchical clustering of E14.5 Rlf-DMRs. [file 12915_2015_128_MOESM3_ESM.pdf]

Supplemental Figure 2

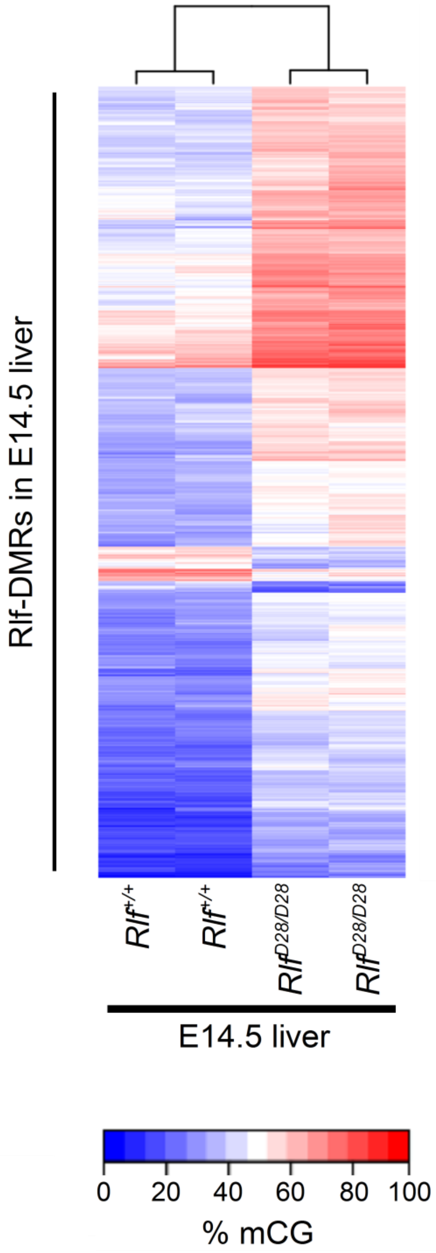

Hierarchical clustering of E14.5 Rlf-DMRs

A total of 1,329 Rlf-DMRs were identified between the livers of *Rlf*$^{MommeD28/MommeD28}$ mice and wild-type. The plot was made from the weighted averages of CpG methylation at each Rlf-DMR.
