# Supplementary material for: The recently identified modifier of murine metastable epialleles, Rearranged L-Myc Fusion, is involved in maintaining epigenetic marks at CpG island shores and enhancers
Source: BMC Biol. 2015 Mar 26;13:21. doi: 10.1186/s12915-015-0128-2 (PMC4381397; doi:10.1186/s12915-015-0128-2)
Supplement: Additional file 5: Figure S3. — Hierarchical clustering of %mCG for random 1 kb regions from the bisulphite converted genomes prepared for this study. [file 12915_2015_128_MOESM5_ESM.pdf]

Supplemental Figure 3

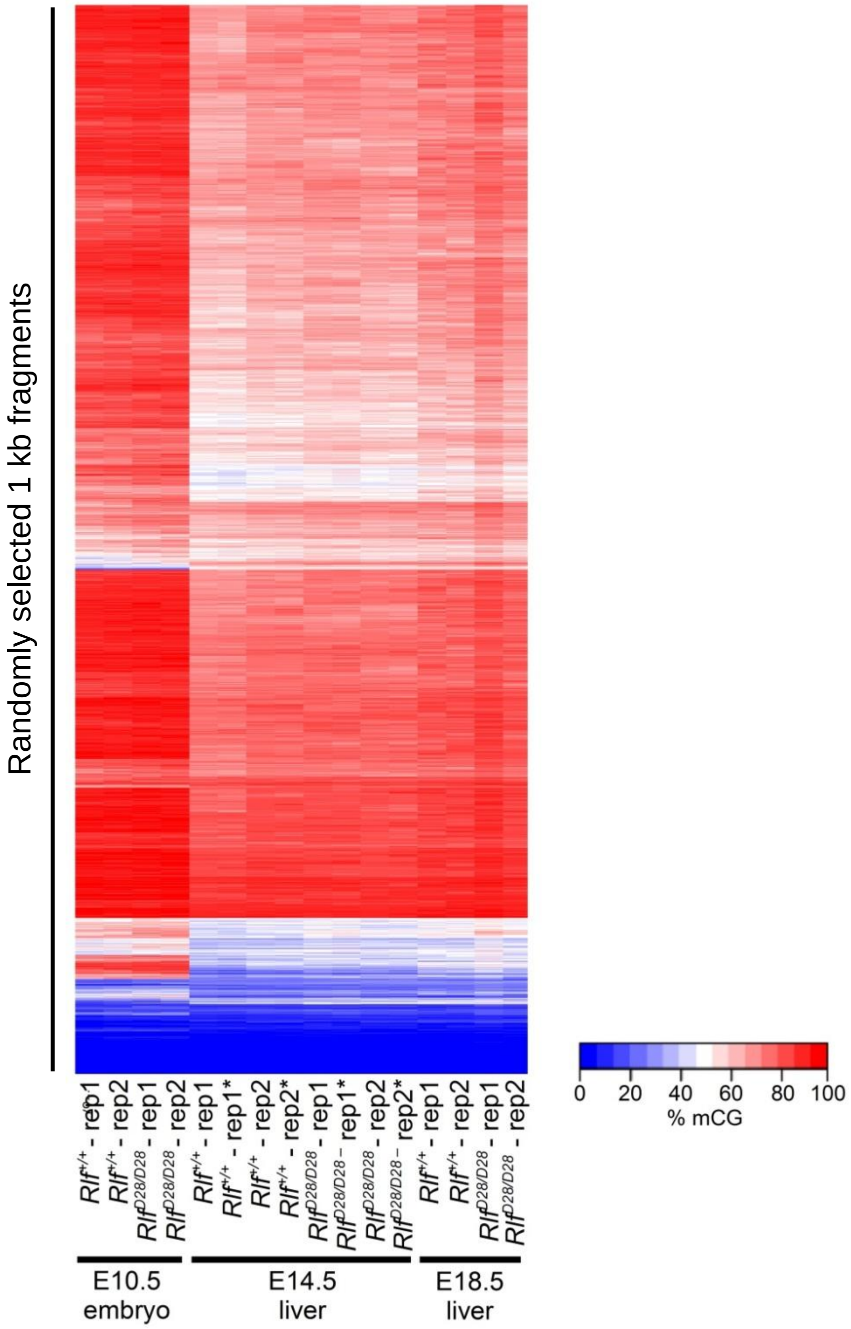

Hierarchical clustering of %mCG for random 1kb regions from the bisulphite converted genomes prepared for this study

Weighted CpG methylation averages for 9000 randomly selected 1kb regions from the mouse genome, each with >7 CpGs and a per-CG read coverage > 5 across all datasets, were plotted. Two wild-type and two *Rlf*$^{MommeD28/MommeD28}$ mice were used for each tissue. The E14.5 livers, initially sequenced to ~30-fold coverage, were sub-sampled (indicated by *) to ~15-fold coverage to allow for direct comparisons with the E10.5 embryo and E18.5 liver datasets (both sequenced to ~15-fold coverage).
