# Supplementary material for: The recently identified modifier of murine metastable epialleles, Rearranged L-Myc Fusion, is involved in maintaining epigenetic marks at CpG island shores and enhancers
Source: BMC Biol. 2015 Mar 26;13:21. doi: 10.1186/s12915-015-0128-2 (PMC4381397; doi:10.1186/s12915-015-0128-2)
Supplement: Additional file 7: Figure S4. — Hierarchical clustering of Rlf-DMRs with normal methylation in at least one tissue. [file 12915_2015_128_MOESM7_ESM.pdf]

Supplemental Figure 4

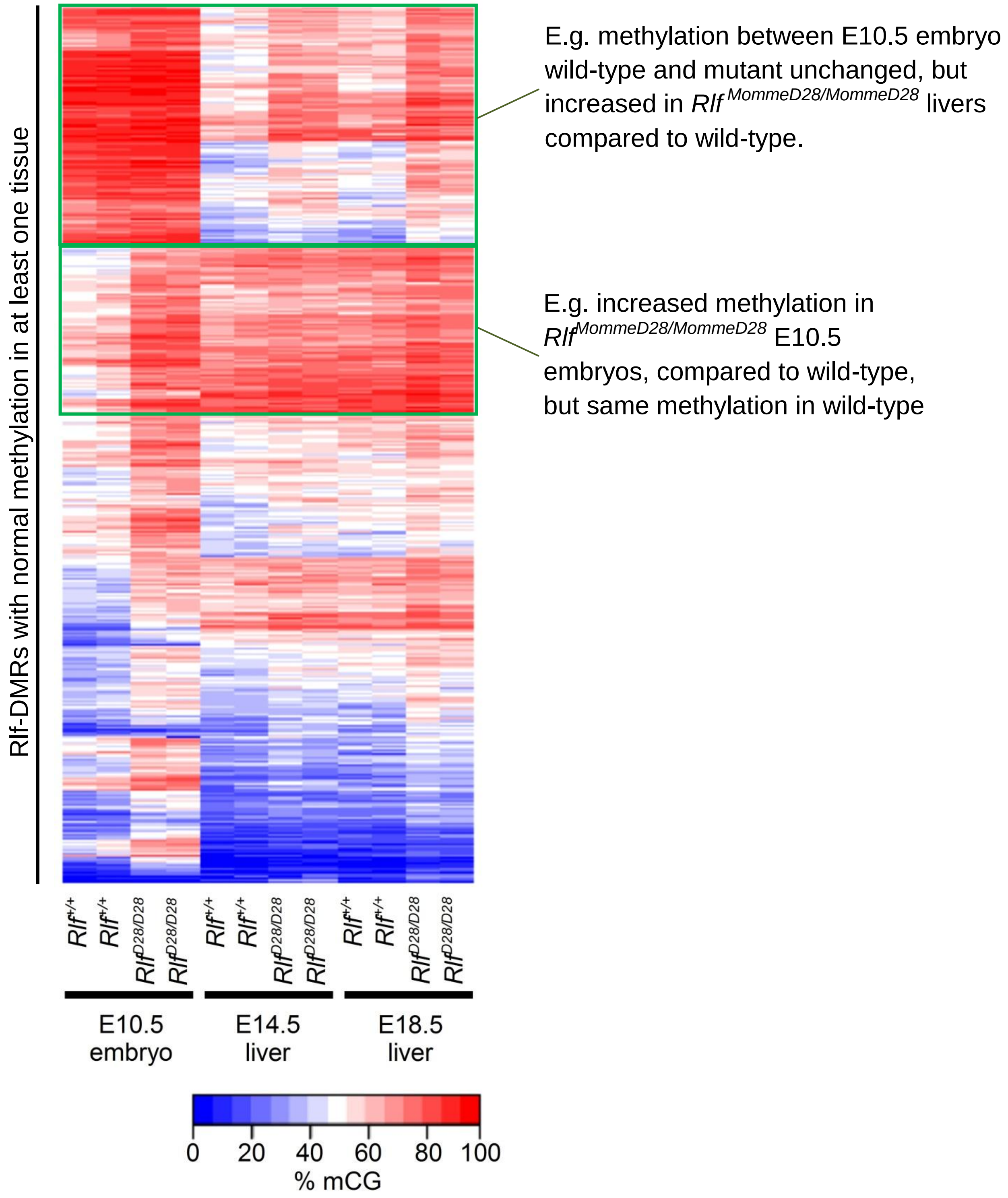

Hierarchical clustering of Rlf-DMRs with normal methylation in at least one tissue

Plot of 986 Rlf-DMRs for which wild-type and *Rlf* $^{MommeD28/MommeD28}$ were differentially methylated in one or two of the three tissues (>15% shift), but with no difference between wild-type and mutant in at least one tissue (<5% shift).
