# Supplementary material for: The recently identified modifier of murine metastable epialleles, Rearranged L-Myc Fusion, is involved in maintaining epigenetic marks at CpG island shores and enhancers
Source: BMC Biol. 2015 Mar 26;13:21. doi: 10.1186/s12915-015-0128-2 (PMC4381397; doi:10.1186/s12915-015-0128-2)
Supplement: Additional file 8: Figure S5. — Hierarchical clustering of Rlf-DMRs detected for only one liver timepoint. [file 12915_2015_128_MOESM8_ESM.pdf]

Supplemental Figure 5

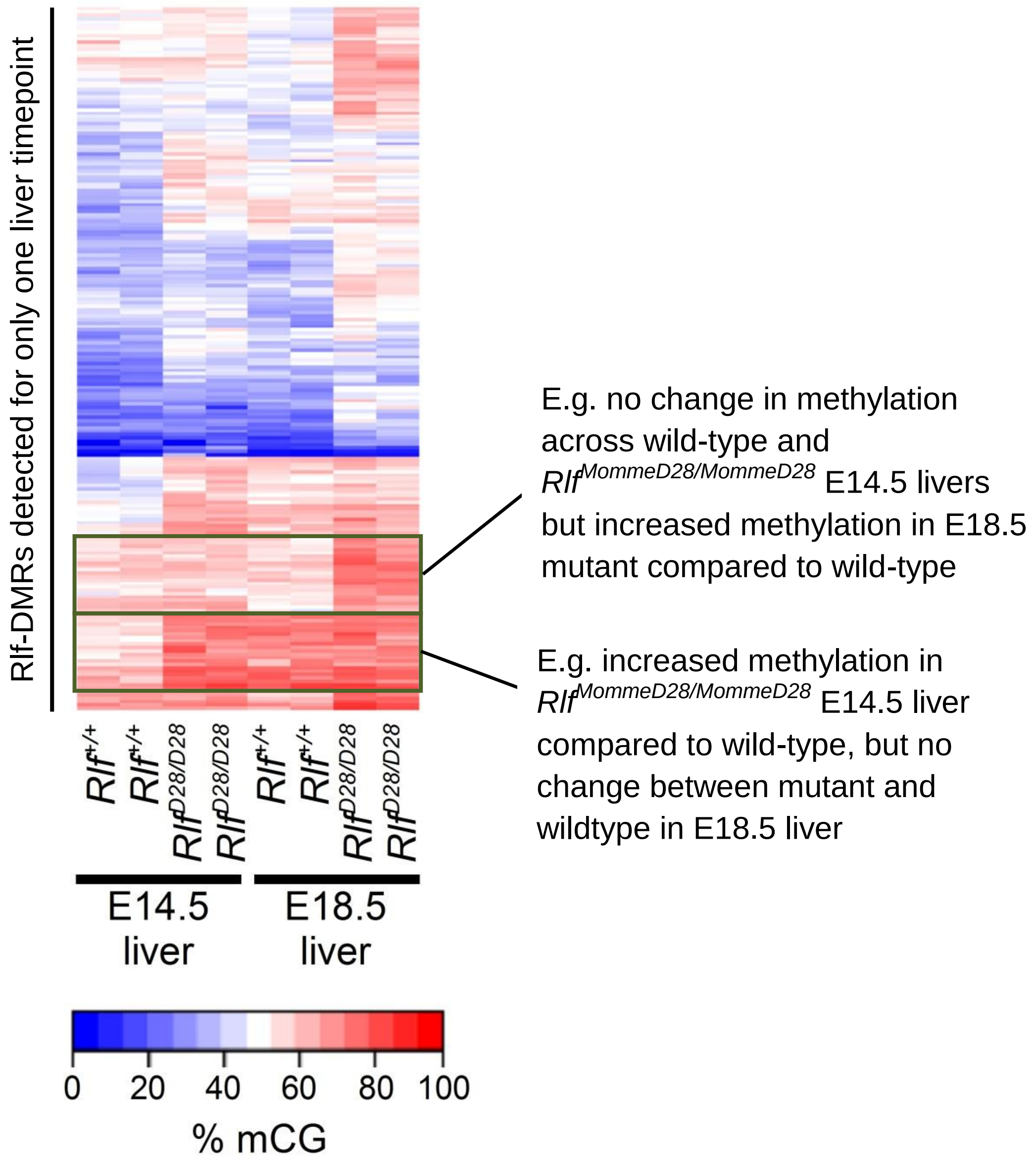

Hierarchical clustering of Rlf-DMRs detected for only one liver timepoint
Plot of 200 Rlf-DMR weighted averages for which methylation differs between wild-type and mutant at one liver timepoint (>15% change in methylation), but not the other (<5% change in methylation).
